# A systematic review and meta-analysis of carbohydrate benefits associated with randomized controlled competition-based performance trials

**DOI:** 10.1186/s12970-016-0139-6

**Published:** 2016-07-11

**Authors:** Martin Pöchmüller, Lukas Schwingshackl, Paolo C. Colombani, Georg Hoffmann

**Affiliations:** Department of Nutritional Sciences, Faculty of Life Sciences, University of Vienna, Althanstraße 14 (UZAII), A-1090 Vienna, Austria; German Institute of Human Nutrition, Arthur-Scheunert-Allee 114-116, D-14558 Nuthetal, Germany; Swiss Federal Institute of Sport Magglingen (SFISM), CH-2532 Magglingen, Switzerland

**Keywords:** Carbohydrate supplementation, Ergogenic effects, Exercise, Meta-analysis, Systematic review

## Abstract

**Background:**

Carbohydrate supplements are widely used by athletes as an ergogenic aid before and during sports events. The present systematic review and meta-analysis aimed at synthesizing all available data from randomized controlled trials performed under real-life conditions.

**Methods:**

MEDLINE, EMBASE, and the Cochrane Central Register of Controlled Trials were searched systematically up to February 2015. Study groups were categorized according to test mode and type of performance measurement. Subgroup analyses were done with reference to exercise duration and range of carbohydrate concentration. Random effects and fixed effect meta-analyses were performed using the Software package by the Cochrane Collaboration Review Manager 5.3.

**Results:**

Twenty-four randomized controlled trials met the objectives and were included in the present systematic review, 16 of which provided data for meta-analyses. Carbohydrate supplementations were associated with a significantly shorter exercise time in groups performing submaximal exercise followed by a time trial [mean difference −0.9 min (95 % confidence interval −1.7, −0.2), *p* = 0.02] as compared to controls. Subgroup analysis showed that improvements were specific for studies administering a concentration of carbohydrates between 6 and 8 % [mean difference −1.0 min (95 % confidence interval −1.9, −0.0), *p* = 0.04]. Concerning groups with submaximal exercise followed by a time trial measuring power accomplished within a fixed time or distance, mean power output was significantly higher following carbohydrate load (mean difference 20.2 W (95 % confidence interval 9.0, 31.5), *p* = 0.0004]. Likewise, mean power output was significantly increased following carbohydrate intervention in groups with time trial measuring power within a fixed time or distance (mean difference 8.1 W (95 % confidence interval 0.5, 15.7) *p* = 0.04].

**Conclusion:**

Due to the limitations of this systematic review, results can only be applied to a subset of athletes (trained male cyclists). For those, we could observe a potential ergogenic benefit of carbohydrate supplementation especially in a concentration range between 6 and 8 % when exercising longer than 90 min.

**Electronic supplementary material:**

The online version of this article (doi:10.1186/s12970-016-0139-6) contains supplementary material, which is available to authorized users.

## Background

Carbohydrates are one of the two main fuels for sport activities and their importance for optimal sport performance both in training and in competition is generally undisputed among experts [1, 2].

Carbohydrates are also used by athletes as an ergogenic aid before and during sport events even when they have repleted carbohydrate reserves. The scientific background of carbohydrates as an ergogenic nutritional supplement has been the subject of numerous investigations with the majority of results indicating a performance-enhancing effect of carbohydrate supplementation shortly before and during a performance bout [3–10].

In some of these studies, subjects were competing in a fasted state. Overnight fasting may probably result in more easily reproducible outcomes due to a more balanced state of metabolism in comparison to a postprandial state [11]. However, athletes intuitively avoid a fasted state before any competition and it is not recommended in the pertinent literature. It has been indicated that during an overnight fast liver glycogen stores are reduced substantially by amounts as high as 80 % [1, 2]. Therefore, suboptimal carbohydrate stores are likely to be present when beginning an exercise in a fasted state. Furthermore, many performance studies used time-to-exhaustion tests, which asses how long subjects can exercise at a given intensity. Again, this protocol does not always reflect the conditions of a real competition because athletes, at least in elite sports, should either perform as fast as possible for a given distance (e.g., races) or as well as possible within a given time (e.g., team sports). Currell and Jeukendrup [12] assessed various performance protocols and concluded that those in which subjects were asked to complete a fixed distance/amount of work as fast as possible or to accomplish as much work/distance as possible in a given time (i.e. time trails), yielded better results with respect to validity, reliability and sensitivity as compared to time-to-exhaustion protocols [12].

In 2013, a systematic review by Colombani and co-workers [11] addressed all these aspects. Their results suggests that only 11 out of 22 investigations included in the review resulted in a significant improvement of performance following carbohydrate supplementation indicating a high amount of uncertainty concerning the benefits of carbohydrate supplementation in field experiments trying to copy a realistic performance setting.

It was the purpose of the present study to expand the approach by Colombani et al. [11] via an updated literature search in order to yield an extended number of suitable studies so that the systematic review can be combined with a statistical synthesis of the available data using a meta-analytical approach.

## Methods

### Search strategy

Data of the original search by Colombani et al. [11] were used as starting point. The authors performed a search in “PubMed” up to September 3, 2011 using the following combination of key words: (Exercise OR Sport OR Athlete OR Athletes) AND (Hydration OR Water OR Fluid OR Drink OR Drinks OR Beverage OR Beverages OR Glycogen OR Loading OR Carbo OR Carbohydrate OR Carbohydrates OR Glucose OR Fructose OR Maltodextrin) NOT (Mice OR Mouse OR Pig OR Pigs OR Rat OR Rats OR Horse OR Horses OR Fish OR Dog OR Dogs OR Patient OR Patients OR Disease OR Diseases OR Diabetes OR Obesity OR Obese OR “Cord injury” OR “Wheelchair).

In addition to the systematic search of Colombani and coworkers [11] we searched the electronic databases “Embase” as well as the “Cochrane Central Register Of Controlled Trials” up to February, 2016 and expanded the search in “PubMed” starting September 4^th^, 2011 to February, 2016 using the same combinations of search terms with the following exceptions: we used “Human” and “Adult <18 to64 years” as further limitations in the database “Embase”. Hand search was done using the reference lists of two meta-analyses [7, 8], yielding one additional article suitable for this systematic review [13].

### Inclusion criteria

In accordance to Colombani et al. [11], the following inclusion criteria were defined:

- Randomized, crossover, placebo-controlled and if possible blinded study design. Blinding was not feasible as an absolute criterion, as sometimes the intervention could not be fully masked;
- Mean age of the subjects between 18 and 40 years, but no restriction with respect to gender;
- A reported VO_2max_ ≥ 50 mL/kg/min (for an appropriate estimation of subject’s fitness level);
- Assessment of body mass;
- Subjects were tested in the postprandial state (between 2 h and 4 h after ingesting last meal);
- Performance test had to be either of a time trial (TT) character or a submaximal exercise followed by a time trial (S + TT);
- For studies with carbohydrate intake immediately prior to and/or during exercise, we included only studies with provision of any type of carbohydrates, electrolytes and water but no further components.

### Exclusion criteria

- Studies with time-to-exhaustion tests or studies with insufficient methodological information to enable a check of the inclusion criteria were excluded.

### Categorization of interventions

To yield more homogeneous study designs it was necessary to categorize the studies by defining comparable interventions prior to statistical analysis. Classification of groups was performed according to test mode (cycling, running, soccer), carbohydrate intervention (carboloading vs. no carboloading; carbohydrate mouth rinse vs. placebo mouth rinse; ingestion of carbohydrate containing drinks vs. drinks containing no carbohydrates), type of intervention (TT or S + TT), and outcome [performance as time needed to cover a fixed distance (or a set amount of work); distance covered within a fixed time, or power accomplished within a fixed time (or fixed distance)]. Taken together, this resulted in the following classification of groups:

- Group 1: Submaximal exercise followed by a time trial measuring time needed to cover a fixed distance or a fixed set amount of work;
- Group 2: Time trial measuring time needed to cover a fixed distance or a fixed set amount of work;
- Group 3: Submaximal exercise followed by a time trial measuring power (W) accomplished within a fixed time or distance;
- Group 4: Time trial measuring power (W) accomplished within a fixed time or distance.

Furthermore, subgroups were formed in order to address two other research questions:

1. Whether the ergogenic effect is dependent on exercise duration (short duration < 90 min vs. long duration > 90 min);
2. If there is an advantage within a specified range of carbohydrate concentrations (6–8 % vs. 1–12 % vs. 12–18 %).

In the scientific literature, the different mechanisms for ergogenic effects of carbohydrates with respect to short and long lasting physical exercise was explained to be due to different carbohydrate availability. For exercise durations lasting ≤ 90 min there should be sufficient substrate without power loss given the condition of regularly filled glycogen stores [14–16]. Therefore we compared exercise durations ≤ and > than 90 min.

### Statistical analyses

Data were analyzed using the Review Manager 5.3 software provided by the Cochrane Collaboration (http://tech.cochrane.org/revman). Differences in means were compared for outlining possible differences between carbohydrates and placebo with a fixed-effect meta-analysis using the inverse-variance method. However, when heterogeneity exceeded the level of 50 %, the random-effects model was used. The Cochrane Collaboration suggests to use meta-analyses in order to synthesize evidence from multiple experiments addressing the same research questions. Checking consistency of the results is of major importance in meta-analyses. Statistical heterogeneity in studies is characterized by 95 % CI that show poor overlap. We used the I^2^ statistic to detect heterogeneity [17]. If considerable heterogeneity is observed (I^2^ ≥ 50 %), fixed-effect models should be avoided, since they underperform in that context. Random effects models provide a more conservative approach yielding better estimates [18].

Descriptive data of included trials are given as mean ± SD. Pooled estimates of the effects size obtained by either comprehensive or subgroup meta-analyses are reported as mean difference together with the 95 % confidence intervals, respectively. *P*-values < 0.05 were considered to be statistically significant. Moreover, effects sizes are given as standardized mean differences (SMD) for each analysis group as Additional files 1, 2, 3 and 4 (see corresponding Result section).

## Results

### Literature search

**Fig. 1:**
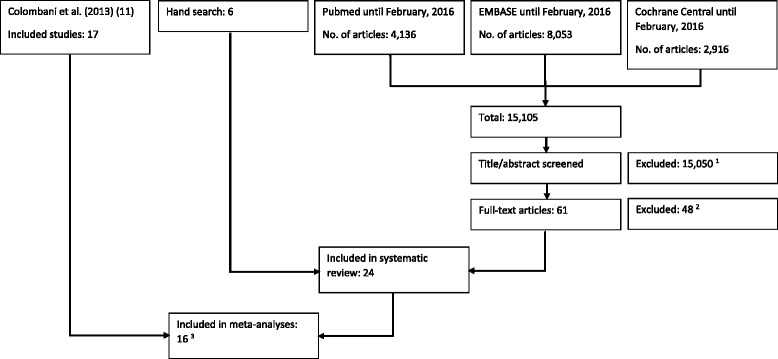
Flow diagram of article selection process. ^1^ Exclusion of duplicates. ^2^ not randomized controlled trials, different age group, no time trial or submaximal exercise followed by time trial. ^3^ Considerable differences with respect to type of exercise: McGawley et al., [29]; Rollo et al., [28]. Soccer-specific protocol: Clarke et al., [30]. Considerable differences with respect to carbohydrate intervention: Burke et al., [25]; Burke et al., [26]; Beelen et al., [27]. Inoperable presentation of data: El-Sayed et al., [33]; Flynn et al., [32]

In the original literature search by Colombani et al. [11] performed in the electronic database Pubmed until September 3^rd^, 2011, 16,658 articles were identified. Our own updated search for literature yielded 15,105 articles (4,136 articles from PubMed published between September 4^th^, 2011 and February, 2016, 2,916 articles from Cochrane Central Register Of Controlled Trials, and 8,053 from Embase, respectively). Articles which contained sufficient information in the title or abstract to identify them as not eligible were discarded, if this was not the case, the full text was consulted. Furthermore, 12 reviews [3–6, 9, 10, 19–24] concerning this topic were hand-searched for eligible studies, however no additional study fulfilling the search criteria was identified. In total, the full text of 205 articles was examined yielding 24 studies that met the inclusion criteria and are displayed in the systematic review (Tables 1 and 2). 16 of these articles provided enough information to allow for a quantitative evaluation. Steps of article search and selection are summarized as a flow chart in Fig. 1.

**Table 1:** General characteristics of randomized controlled trials included in the systematic review

Reference	Type	Test	Mode	Test time	CHO content of pre-exercise meal (g/kg body weight)	Drink type during test	Drink during test per h	
							Fluid	CHO
Acker-Hewitt et al., 2012 [38]	CHO vs. W	S + TT	Cycle	20 min + 44 min	1.3	8 % CHO not specified	0.7 L	56 g
Angus et al., 2000 [44]	CHO vs. W	TT	Cycle	166 min	2.8	6 % CHO not specified	1.0 L	60 g
Beelen et al., 2009 [27]^a^	Mouth rinse	TT	Cycle	68 min	2.4	6.4 % MAL	0.0 L	0 g
Burke et al., 2000 [25]^a^	Carboloading	TT	Cycle	148 min	2	Both trials same 7 % GLUP	1.1 L	72 g
Burke et al., 2002 [26]^a^	Carboloading	S + TT	Cycle	120 + 25 min	2	Both trails same 6 % CHO, CHO not specified	0.7 L	44 g
Baur et al., 2014 [39]	CHO vs. W	S + TT	Cycle	120 + 52 min	no data	a) 12 % GLU + FRU (2:1) b) 8 % GLU c) 12 % GLU	0.8 L	a) 93 g b) 62 g c) 93 g
Campbell et al., 2008 [34]	CHO vs. W	S + TT	Cycle	a) 80 + 17 min b) 80 + 17 min c) 80 + 17 min	male: 1.4 female: 1.6	All 5.9 % a) SUC + GLU + FRU drink b) MAL + FRU gel c) SUC + GLU sport beans	0.7 L	43 g
Clarke et al., 2011 [30]^a^	CHO vs. W	S + TT	Soccer	90 + 3 min	no data	6.6 % CHO not specified	0.9 L	59 g
Cox et al., 2008 [35]	CHO vs. W	S + TT	Cycle	100 min + 30 min	2.1	10 % GLU	1.125 L	112.5 g
Cox et al., 2010 [36]	CHO vs. W	S + TT	Cycle	100 min + 30 min	2.1	10 % GLU	1.125 L	112.5 g
Desbrow et al., 2004 [45]	CHO vs. W	TT	Cycle	63 min	2	6 % CHO not specified	1.0 L	61 g
El-Sayed et al., 1995 [33]^a^	CHO vs. W	S + TT	Cycle	60 + 10 min	no data	7.5 % GLU	0.7 L	54 g
El-Sayed et al., 1997 [47]	CHO vs. W	TT	Cycle	60 min	no data	8 % GLU	0.3 L	25 g
Flynn et al., 1989 [32]^a^	CHO vs. W	S + TT	Cycle	105 + 15 min	3.5	7.7 % GLUP & SUC	0.7 L	58 g
Ganio et al., 2010 [31]	CHO vs. W	S + TT	Cycle	120 + 15 min	no data	6 % CHO not specified	0.9 L	53 g
Hulston et al., 2009 [37]	CHO vs. W	S + TT	Cycle	120 + 59 min	no data	6 % GLU & FRU (2:1)	0.8 L	45 g
Hunter et al., 2002 [46]	CHO vs. W	TT	Cycle	150 min	no data	7 % CHO not specified	0.6 L	42 g
Jeukendrup et al., 2008 [22]	CHO vs. W	TT	Cycle	26 min	no data	6 % SUC & GLU (3:2)	1.2 L	70 g
Langenfeld et al., 1994 [40]	CHO vs. W	TT	Cycle	241 min	no data	7 % MAL & FRU (5:2)	0.5 L	37 g
McGawley et al., 2012 [29]^a^	CHO vs. W	S + TT	Run	88 min + 40 min	no data	14.4 % MAL + FRU (2:1)	0.8 L	115 g
Mitchell et al., 1989 [13]	CHO vs. W	S + TT	Cycle	105 + 15 min	0.7	a) 6 % GLUP & SUC (2:1) b) 12 % GLUP & FRU (2.4:1) c) 18 % GLUP & FRU (4.1:1)	0.6 L	a) 37 g b) 75 g c) 111 g
Nassif et al., 2014 [41]	CHO vs. W	TT	Cycle	135 min	no data	6 % CHO not specified	0.63 L	38 g
Rollo et al., 2010 [28]^a^	CHO vs. W	TT	Run	60 min	2.5	6.4 % CHO not specified	0.4 L	28 g
van Essen et al., 2006 [42]	CHO vs. W	TT	Cycle	135 min	no data	6 % SUC	1.0 L	60 g

*CHO* carbohydrates, *GLU* glucose, *GLUP* glucose polymer, *FRU* fructose, *MAL* maltodextrin, *SUC* sucrose, *S + TT* submaximal exercise + time trial, *TT* time trial, *W* water

^a^not suitable for meta-analyses

**Table 2:** Characteristics of participants in studies eligible for systematic review

Reference	Number of subjects	Gender	Age	VO2max (mL/kg body mass/min)
Acker-Hewitt et al., 2012 [38]	10	Males	28	66
Angus et al., 2000 [44]	8	Males	22	65
Beelen et al., 2009 [27]^a^	14	Males	24	68
Burke et al., 2000 [25]^a^	7	Males	28	64
Burke et al., 2002 [26]^a^	8	Males	28	69
Baur et al., 2014 [39]	8	Males	25	62
Campbell et al., 2008 [34]	16	8 males/8 females	35/32	59/50
Clarke et al., 2011 [30]^a^	12	Males	25	61
Cox et al., 2008 [35]	16	Males	31	65
Cox et al., 2010 [36]	16	Males	31	65
Desbrow et al., 2004 [45]	9	Males	30	65
El-Sayed et al., 1995 [33]^a^	9	Males	24	61
El-Sayed et al., 1997 [47]	8	Males	25	67
Flynn et al., 1989 [32]^a^	7	Males	29	62
Ganio et al., 2010 [31]	14	Males	27	60
Hulston et al., 2009 [37]	10	Males	28	62
Hunter et al., 2002 [46]	8	Males	24	65
Jeukendrup et al., 2008 [22]	12	Males	19	66
Langenfeld et al., 1994 [40]	14	Males	21	56
McGawley et al., 2012 [29]^a^	10	6 males/4 females	26/24	63/62
Mitchell et al., 1989 [13]	10	Males	24	63
Nassif et al., 2014 [41]	10	Males	26	71
Rollo et al., 2010 [28]^a^	10	Males	34	62
van Essen et al., 2006 [42]	10	Males	24	63

^a^not suitable for meta-analyses

### Types of studies

#### Carbohydrate intervention

Two studies were carboloading interventions, one [25] using a TT as the performance test, the other one [26] a submaximal exercise followed by a TT.

We found one eligible study [27] with a mouth-rinse intervention, the remaining 21 studies compared the effect of a carbohydrate-containing drink versus a non-carbohydrate placebo. In eight of these interventions, the carbohydrate type was not specified with only the total amount of carbohydrate being reported. In the remaining 13 studies, either glucose, a glucose-polymer, maltodextrin, fructose, and/or sucrose was used as carbohydrate sources with a concentration ranging between 5.9 and 18 %.

#### Test mode

Most studies used cycling as their exercise mode with three exceptions: Rollo and Williams [28] measured performance while running a distance within a fixed time using a submaximal exercise followd by a TT, McGawley et al. [29] measured performance via running-time needed to cover a fixed distance using only TT, and Clarke et al. [30] investigated the ergogenic effect of carbohydrates with a soccer-specific mode.

Eleven studies used a TT as their performance test with test durations between 26 min to 241 min.

The remaining 13 investigations used a submaximal exercise followed by a TT with test durations between 20 + 44 min-120 + 59 min. Intervention and test modes for all studies are summarized in Tables 1 and 2, respectively.

All of the 16 studies provided enough information for a quantitative evaluation used cycling as their exercise mode. For reason of a better comparability, these studies were assigned to one of four different groups as described in the Methods section. Study designs with both time and power outcomes where assigned to all applicable groups. Two articles presented their outcomes as work [13, 31], which was converted into power prior to analyses by dividing work by the required time.

Results for group 1 and 3 were subdivided based on the administered carbohydrate concentrations, results for group 2 and 4 were subdivided based on exercise duration.

#### Exclusion of studies

Two studies tested the advantage of carbohydrates during a running exercise and were not included into one of the four groups because of considerable physiological differences between this and the other types of exercise [28, 29]. In addition, the study by Clarke et al. [30] was the only eligible study using a soccer-specific protocol and could therefore not be included in the meta-analysis. Other studies had to be excluded due to either different carbohydrate intervention [mouth rinse [27], carboloading [25, 26]], or presentation of data in an inoperable unit (N/m) [32], or presentation of results only via graphics, respectively [33].

### Characteristics of subjects

Subjects were male with the exception of two studies [29, 34] enrolling both genders. Sample size varied between seven and 16 volunteers, mean age ranged between 19 and 35 years, and mean VO_2max_ ranged between 50 and 71 ml/kg body weight/min.

### Performance outcomes

**Fig. 2:**
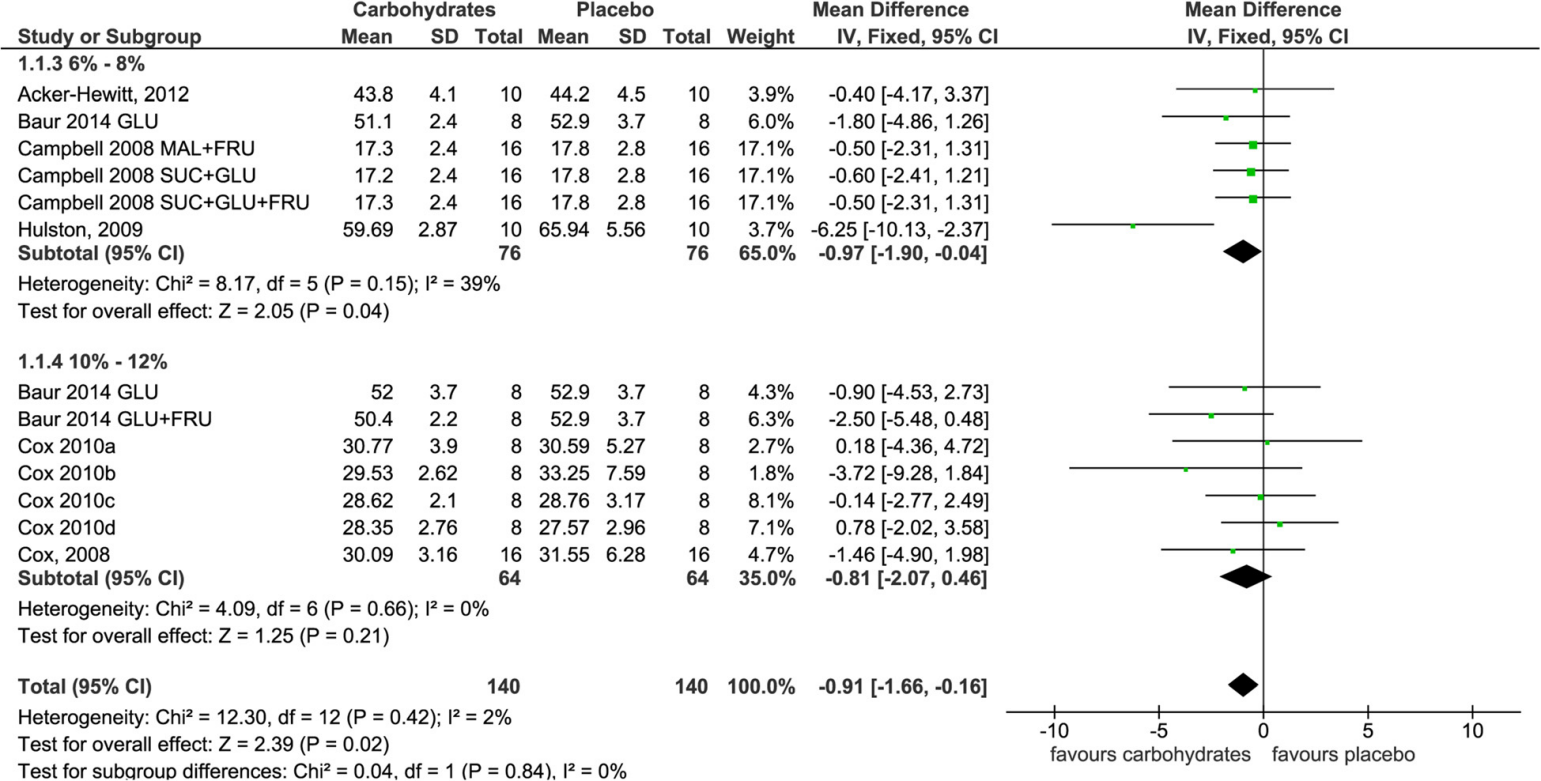
Effects of carbohydrate interventions as compared to placebo on time required to finish a time trial. Forest plot shows pooled mean differences with 95 % confidence intervals (CI) for 6 randomized controlled trials. Subgroup analyses show the results for carbohydrate concentrations ranging between 6–8 % and 10–12 %, respectively. The diamond at the bottom of the graph and the subgroups represents the pooled mean difference with the 95 % CI for all trials following fixed effect meta-analyses. GLU = glucose; FRU = fructose; MAL = maltodextrin; SUC = sucrose

**Fig. 3:**
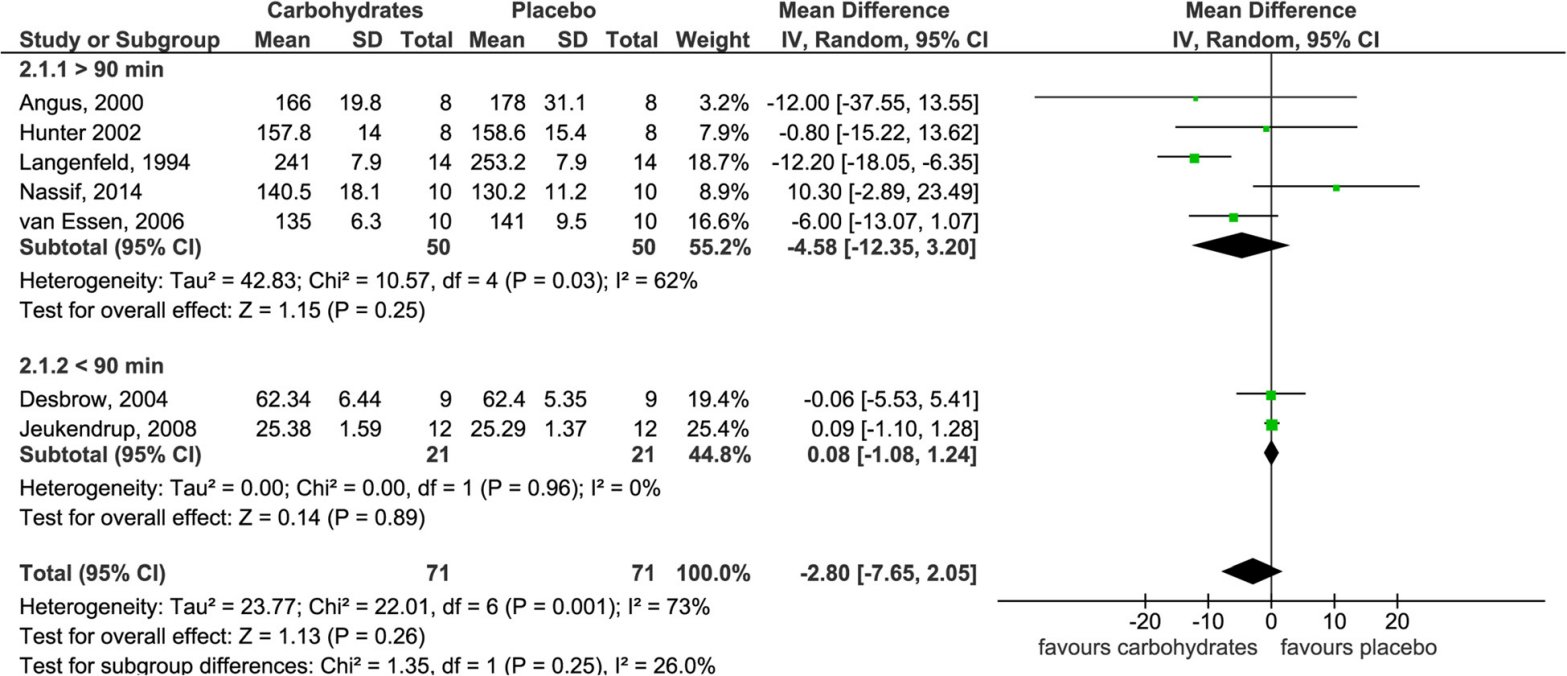
Effects of carbohydrate interventions as compared to placebo on time required to finish a time trial. Forest plot shows pooled mean differences with 95 % confidence intervals (CI) for 7 randomized controlled trials. Subgroup analyses show the results for exercise duration shorter than 90 min or longer than 90 min, respectively. The diamond at the bottom of the graph and the subgroups represents the pooled mean difference with the 95 % CI for all trials following random effects meta-analyses

**Fig. 4:**
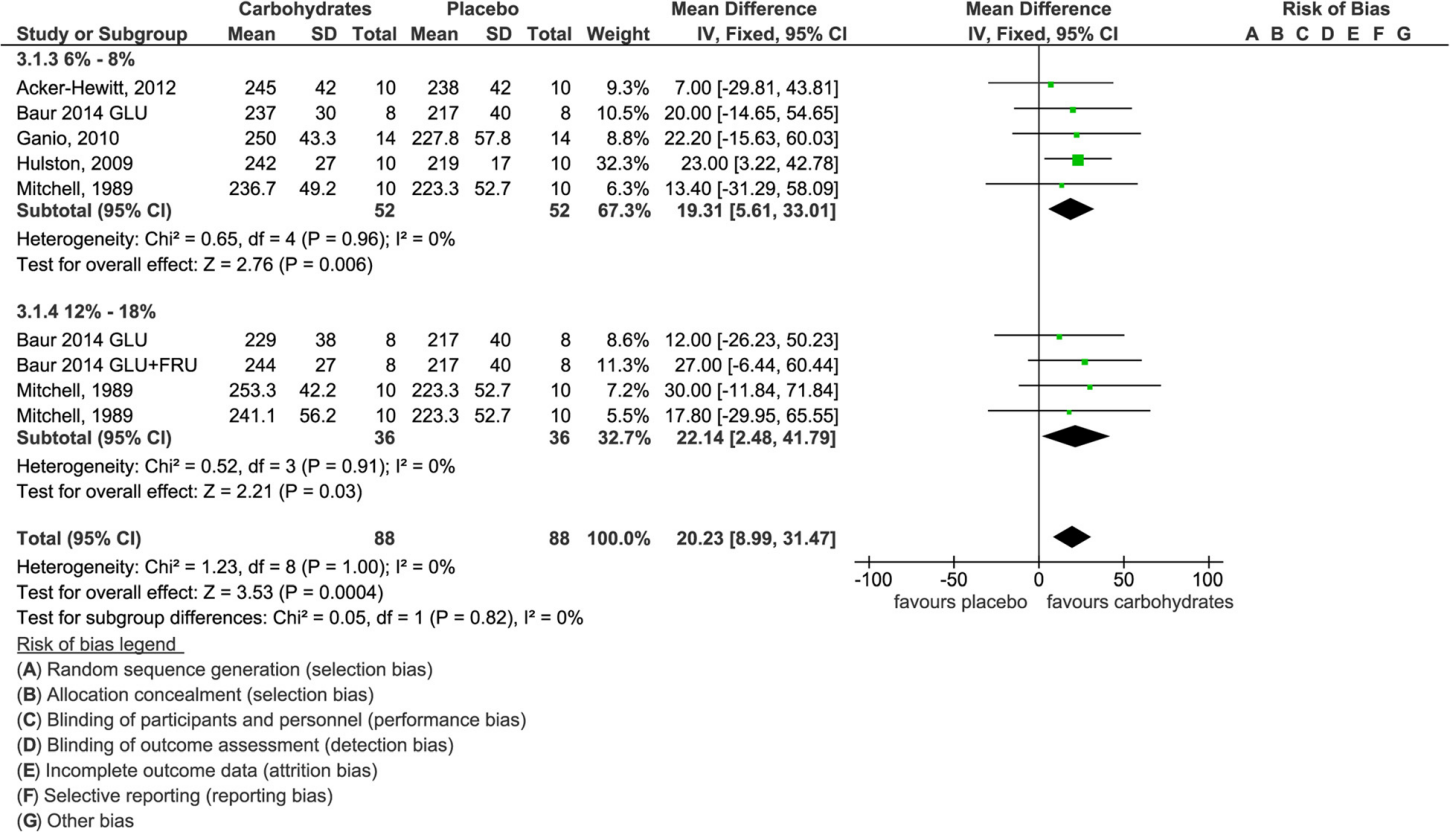
Effects of carbohydrate interventions as compared to placebo on mean power output. Forest plot shows pooled mean differences with 95 % confidence intervals (CI) for 5 randomized controlled trials. Subgroup analyses show the results for carbohydrate concentrations ranging between 6–8 % and 12–18 %, respectively. The diamond at the bottom of the graph and the subgroups represents the pooled mean difference with the 95 % CI for all trials following fixed effect meta-analyses. GLU = glucose; FRU = fructose

**Fig. 5:**
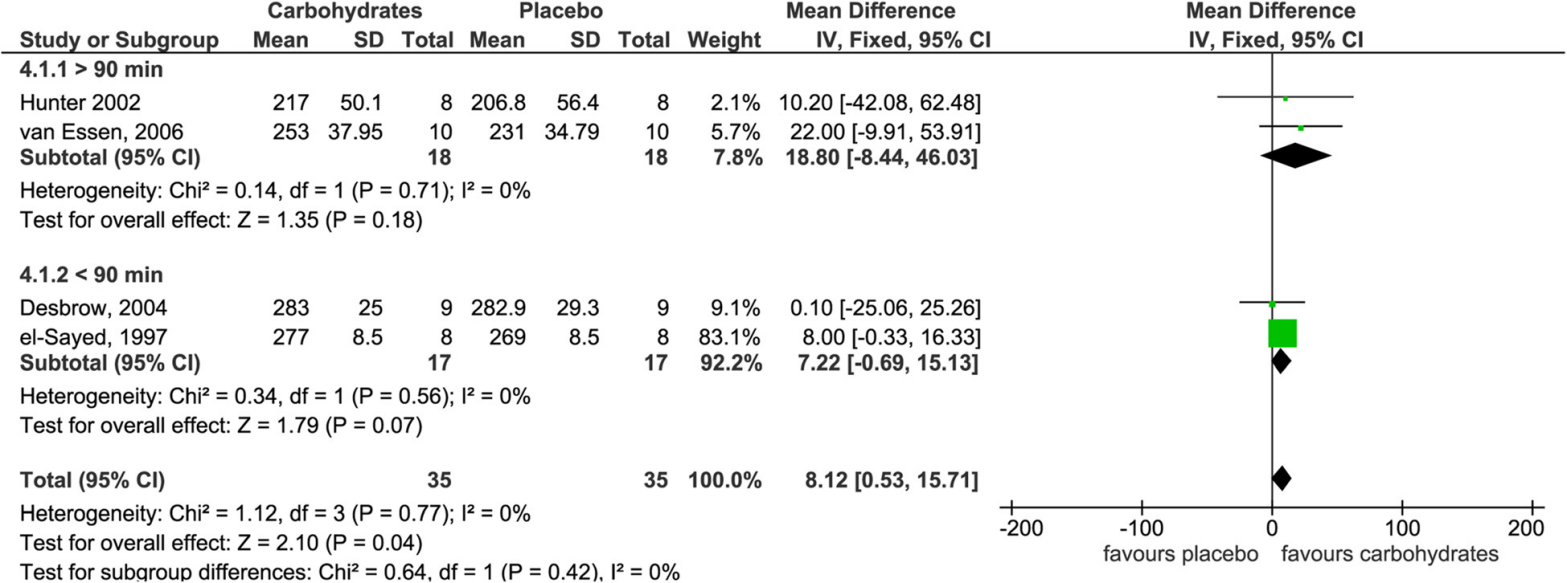
Effects of carbohydrate interventions as compared to placebo on mean power output. Forest plot shows pooled mean differences with 95 % confidence intervals (CI) for 4 randomized controlled trials. Subgroup analyses show the results for exercise duration shorter than 90 min or longer than 90 min, respectively. The diamond at the bottom of the graph and the subgroups represents the pooled mean difference with the 95 % CI for all trials following fixed effect meta-analyses

For each of the four groups, results of both comprehensive as well as subgroup meta-analyses are given in Figs. 2, 3, 4 and 5, respectively. Please note that classification into subgroups was performed for every group independent of resulting numbers of studies.

Group 1 included six studies [34–39] with 13 interventions in total. Pooled estimates of the effects size for the effects of carbohydrate interventions as compared to placebo on time required to finish a TT are presented in Fig. 2 (forest plot showing pooled SMD is given as Additional file 1). Carbohydrate interventions were associated with a significantly lower amount of time [mean differences −0.9 min (95 % CI −1.7, −0.2), *p* = 0.02]. Following subgroup analyses, significant performance improvements remained only for those studies administering a concentration of carbohydrates between 6 and 8 % [MD = −1.0 min (95 % CI −1.9, −0.0), *p* = 0.04].

Group 2 included seven studies [40–46] with seven interventions in total. Figure 3 summarizes the pooled estimates for effect size obtained by a random effects model due to the considerable heterogeneity between studies (I^2^ = 73 %; *P* = 0.001) (forest plot showing pooled SMD is given as Additional file 2). Average cycling time was faster in subjects ingesting carbohydrates as compared to placebo, however, without being statistically significant [mean difference 2.8 min (95 % CI −7.7, 2.1), *p* = 0.26]. Subgroup analysis including only studies with an exercise duration shorter than 90 min revealed a marginally higher average cycling time in the carbohydrate groups [mean difference 0.1 min (95 % CI −1.1, 1.2), *p* = 0.89]. In contrast, subgroup analysis taking into account studies with an exercise duration longer than 90 min resulted in a decreased average cycling time following carbohydrate ingestion when compared to placebo [mean difference −4.6 min (95 % CI −12.4, 3.2), *p* = 0.25].

Group 3 included five studies [13, 31, 37–39] with nine interventions in total, results of which are summarized in Fig. 4 (forest plot showing pooled SMD is given as Additional file 3). Mean power output was significantly more pronounced in participants subjected to a carbohydrate load as compared to placebo [mean difference 20.2 W (95 % CI 9.0, 31.5, *p* = 0.0004]. Comparable results could be obtained following subgroup analyses subclassifying carbohydrate interventions into ranges of 6–8 % [mean difference 19.3 W (95 % CI 5.6, 33.0), *p* = 0.006] and 12–18 % [mean difference 22.1 W (95 % CI 2.5, 41.8), *p* = 0.03], respectively.

Group 4 included four studies [42, 45–47] with four interventions in total. Meta-analytical data are depicted in Fig. 5 (forest plot showing pooled SMD is given as Additional file 4). Mean power output turned out to be significantly increased in volunteers following a carbohydrate intervention [mean difference 8.1 W (95 % CI 0.5, 15.7), *p* = 0.04]. Concerning subgroup analysis, performance tended to be higher in both studies with an exercise duration greater than 90 min [mean difference 18.8 W (95 % CI −8.4, 46.0), *p* = 0.18] and shorter than 90 min [mean difference 7.2 W (95 % CI −0.6, 15.1), *p* = 0.07] without yielding statistically significant results.

## Discussion

Based upon the recent systematic review by Colombani et al. [11], it was the purpose of the present study to synthesize all available data from randomized controlled trials investigating the potential ergogenic effects of carbohydrate supplementation via meta-analysis. Due to the in-between heterogeneity of trials with respect to study design, we decided to evaluate only studies choosing cycling as the mode of exercise. Moreover, four groups of carbohydrate interventions with respect to test and performance measurement were classified in order to achieve a better comparability of results. Taken together, all four groups indicated an improved performance following carbohydrate intervention as compared to placebo with differences being statistically significant in group 1 (submaximal exercise followed by a time trial measuring time needed to cover a fixed distance or a fixed set amount of work), group 3 (submaximal exercise followed by a time trial measuring power (W) accomplished within a fixed time or distance), and group 4 (time trial measuring power (W) accomplished within a fixed time or distance), respectively.

### Subgroups duration

Duration of exercise ≤ 90 min did not result in statistically significant differences between carbohydrate interventions and placebo either in group 2 or in group 4. These findings seem to be in contrast with studies reporting an improved performance via carbohydrate mouth rinsing [10, 48–54]. It has been suggested that oral receptors within the mouth and the digestive tract sense carbohydrates and activate brain regions associated with reward and pleasure which may lead to enhanced performance [5, 10, 48]. However, most mouth rinse studies were conducted in a fasted state [48, 50, 52, 53] or had other limitations such as lack of or improper randomization [51, 54] or uncertain time of last ingested meal [49]. In our systematic review, three studies [27, 33, 38] with an exercise duration less than 90 min could not be included in either groups 2 or group 4. Beelen et al. [27] demonstrated a non-significant performance decline when testing a carbohydrate mouth rinse. Likewise, Acker-Hewitt et al. [38] did not find a significantly better performance subsequent to a carbohydrate solution when compared to placebo, while El-Sayed et al. [33] could detect an increase in performance capacity. Therefore, it seems premature to finally evaluate the potential benefit of ingesting carbohydrates in short-term exercises (less than 90 min), further trials reflecting realistic conditions are necessary.

Subgroup analysis of five trials with a duration time higher than 90 min in group 2 resulted in a trend towards a decreased time needed to cover a fixed distance or a fixed set amount of work. A similar trend could be observed in group 4, albeit with only two trials included in the subgroup. Taking all results under consideration, a performance benefit through carbohydrates might be possible when exercise duration exceeds 90 min. However, similar to subgroups with ≤ 90 min, additional studies are required for evidence-based recommendations.

### Subgroups carbohydrate concentration

Irrespective of specific carbohydrate concentrations, meta-analytical results of both groups 1 and 3 yielded statistically significant benefits for carbohydrate supplementation. In general, this might be due to multiple factors including maintenance of blood glucose [55–57] and high levels of carbohydrate oxidation especially towards the end of exercise [58, 59], thus sparing liver glycogen [60–63], as well as a central effect of carbohydrates [48, 52].

Regarding range of carbohydrate concentrations, results were statistically significant in favour of the lower range of 6–8 % of carbohydrate supplementation both in groups 1 and 3, while corresponding results for the higher range was only significant in group 3 (12–18 %), but not in group 1 (10–12 %). However, in both groups, the respective higher concentration range resulted in greater statistical variance when compared to the 6–8 % range. Therefore, one might speculate an impact of the administered carbohydrate type becoming more effective at higher concentrations. A high dose of ingested carbohydrates while exercising may cause gastrointestinal discomfort [64] which subsequently may decrease performance [65]. The maximal rate at which a single type of ingested carbohydrate can be oxidized is 60–70 g/h and ingesting more than this amount will not augment the oxidation rate but rather increases the chance for gastrointestinal discomfort [22]. It has been suggested that the ingestion of carbohydrates that use different, not competing, transporters increases the maximal carbohydrate oxidation rate (up to 105 g/h) [66], which has been verified by numerous studies [24, 39, 67–69]. In the study by Baur and co-workers [39], three different carbohydrate solutions were examined (8 % glucose solution, 12 % glucose solution, 12 % glucose–fructose (2:1) solution). The glucose-fructose solution achieved the greatest performance improvement, while the 12 % glucose solution did not affect performance significantly. Likewise, three different carbohydrate solutions (a 6 % glucose polymer-sucrose-solution (2:1, 37 g/h), a 12 % glucose polymer-fructose solution (2.4:1, 75 g/h), and an 18 % (4.1:1, 111 g/h) glucose polymer-fructose solution) were comparatively investigated in the trial by Mitchell et al. [13]. The best performance outcome was found with the 12 % glucose polymer-fructose solution. Despite no direct measurement of gastrointestinal symptoms, the authors concluded that the 18 % solution caused gastrointestinal distress and therefore the performance enhancement was not as high as with the 12 % solution [13].

Thus, the carbohydrate concentration resulting in optimal performance seems to be dependent on many factors, although our data suggests a more consistent benefit with carbohydrate solutions ranging between 6 and 8 %.

### Strengths and limitations

The protocol of the present systematic review was designed to summarize the available evidence on the ergogenic effects of carbohydrate supplementation as an expansion of the results by Colombani et al. [11] focusing on randomized controlled trials investigating the outcomes of their interventions under real-life conditions (no overnight fasting, no time-to-exhaustion tests). Moreover, we decided to categorize trials with respect to types of test and performance measurements. This rigid protocol allows for better comparison between the different trials, it is associated with a number of limitations as well. First of all, the number of studies suitable for meta-analyses turned out to be rather low. All of the 16 trials providing extractable data for meta-analyses used cycling as their exercise mode. Although this might be another aspect increasing the homogeneity of the results, it is not possible to draw any conclusions for other types of exercise such as running. Data on the content of the last meal prior to trials suggest heterogeneous pre-exercise carbohydrate intake between studies. Another common limitation of performance studies is the only low to average power with respect to the number of participants ranging between 16 and 32 volunteers in the present meta-analyses. Since only one trial [39] enrolled subjects with a mean VO_2max_ that would classify them as elite endurance athletes, the results are most likely not affected by heterogeneity between baseline capacities of study participants. In addition, with the exception of References [29] and [34], all trials were performed with male volunteers hampering transfer of results to female athletes. Following conversion of absolute values into percentage data, results were widely spread yielding improvements in assessed outcomes between 0.2 % [45] –13 % [13] as well as declines ranging between −0.6 % [22] and −7.3 % [41], respectively. This may serve as a potential indicator for the heterogeneous study designs.

## Conclusions

In conclusion there may be a benefit for trained male cyclists when ingesting carbohydrates in a concentration range of 6–8 % just before and/or while exercising longer than 90 min. Due to lack of sufficient data, it is difficult to extrapolate this result to elite or generally female athletes. Moreover, further research is needed to gain additional information on exercise durations lower than 90 min and in a wider variety of types of exercise.

## Abbreviations

CHO, carbohydrates; CI, confidence interval; FRU, fructose; GLU, glucose; GLUP, glucose polymers; MAL, maltodextrin; MD, mean difference; S + TT, submaximal exercise followed by time trial; SUC, sucrose; TT, time trial

## Additional files

Additional file 1: Figure S1. Effects of carbohydrate interventions as compared to placebo on time required to finish a time trial. Forest plot shows pooled standardized mean differences with 95 % confidence intervals (CI) for 6 randomized controlled trials. Subgroup analyses show the results for carbohydrate concentrations ranging between 6–8 % and 10–12 %, respectively. The diamond at the bottom of the graph and the subgroups represents the pooled mean difference with the 95 % CI for all trials following fixed effect meta-analyses. GLU = glucose; FRU = fructose; MAL = maltodextrin; SUC = sucrose. Title: File format: tiff (TIF 7950 kb) Additional file 2: Figure S2. Effects of carbohydrate interventions as compared to placebo on time required to finish a time trial. Forest plot shows pooled standardized mean differences with 95 % confidence intervals (CI) for 7 randomized controlled trials. Subgroup analyses show the results for exercise duration shorter than 90 min or longer than 90 min, respectively. The diamond at the bottom of the graph and the subgroups represents the pooled mean difference with the 95 % CI for all trials following random effects meta-analyses. (TIF 6850 kb) Additional file 3: Figure S3. Effects of carbohydrate interventions as compared to placebo on mean power output. Forest plot shows pooled standardized mean differences with 95 % confidence intervals (CI) for 5 randomized controlled trials. Subgroup analyses show the results for carbohydrate concentrations ranging between 6–8 % and 12–18 %, respectively. The diamond at the bottom of the graph and the subgroups represents the pooled mean difference with the 95 % CI for all trials following fixed effect meta-analyses. GLU = glucose; FRU = fructose. (TIF 7339 kb) Additional file 4: Figure S4. Effects of carbohydrate interventions as compared to placebo on mean power output. Forest plot shows pooled standardized mean differences with 95 % confidence intervals (CI) for 4 randomized controlled trials. Subgroup analyses show the results for exercise duration shorter than 90 min or longer than 90 min, respectively. The diamond at the bottom of the graph and the subgroups represents the pooled mean difference with the 95 % CI for all trials following fixed effect meta-analyses. (TIF 5891 kb)
