# Supplementary figures and images for: A systematic review and meta-analysis of carbohydrate benefits associated with randomized controlled competition-based performance trials
Source: J Int Soc Sports Nutr. 2016 Jul 11;13:27. doi: 10.1186/s12970-016-0139-6 (PMC4940907; doi:10.1186/s12970-016-0139-6)

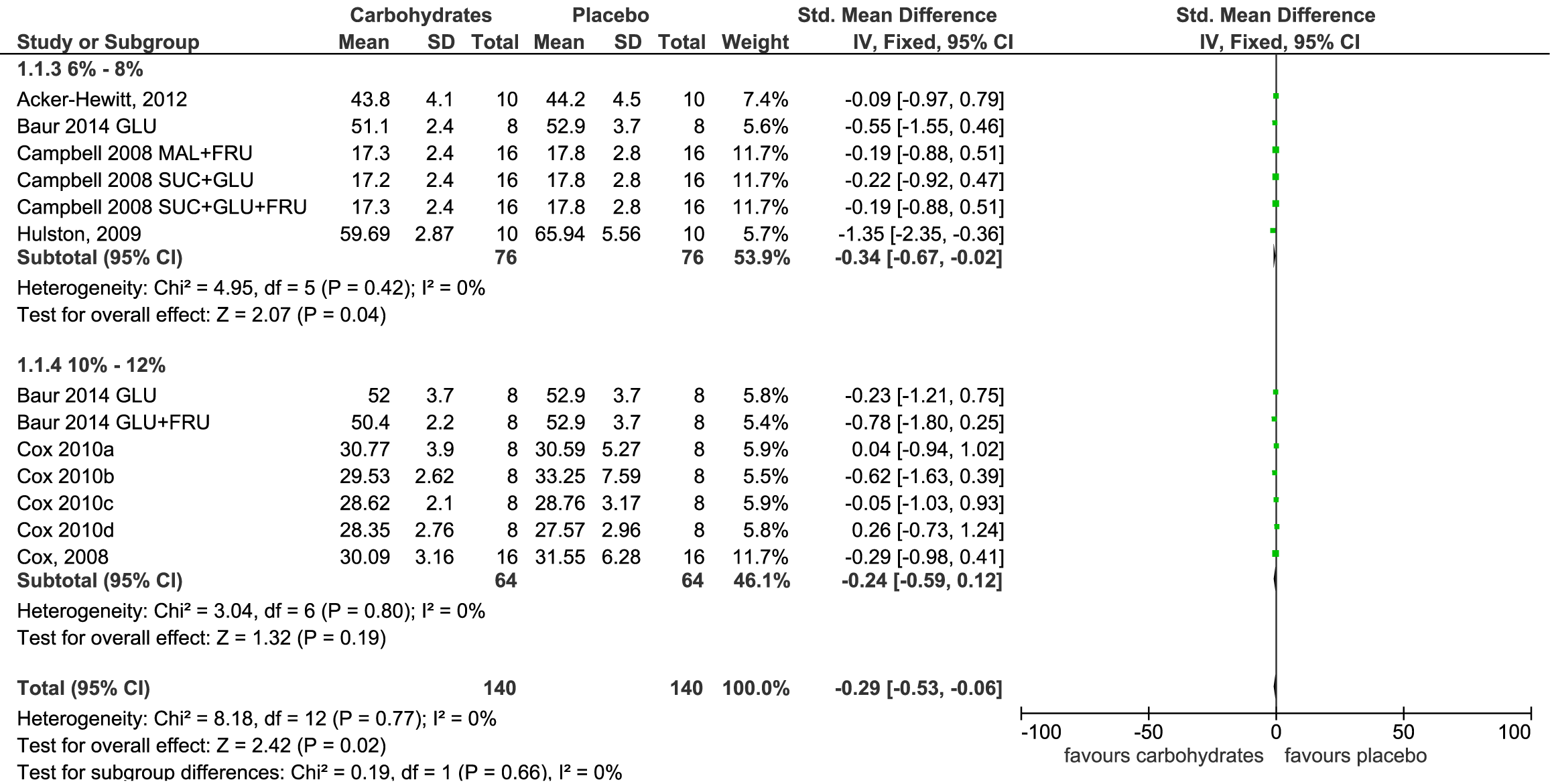

Supplement: Additional file 1: Figure S1. — Effects of carbohydrate interventions as compared to placebo on time required to finish a time trial. Forest plot shows pooled standardized mean differences with 95 % confidence intervals (CI) for 6 randomized controlled trials. Subgroup analyses show the results for carbohydrate concentrations ranging between 6–8 % and 10–12 %, respectively. The diamond at the bottom of the graph and the subgroups represents the pooled mean difference with the 95 % CI for all trials following fixed effect meta-analyses. GLU = glucose; FRU = fructose; MAL = maltodextrin; SUC = sucrose. Title: File format: tiff (TIF 7950 kb) [file 12970_2016_139_MOESM1_ESM.tif]

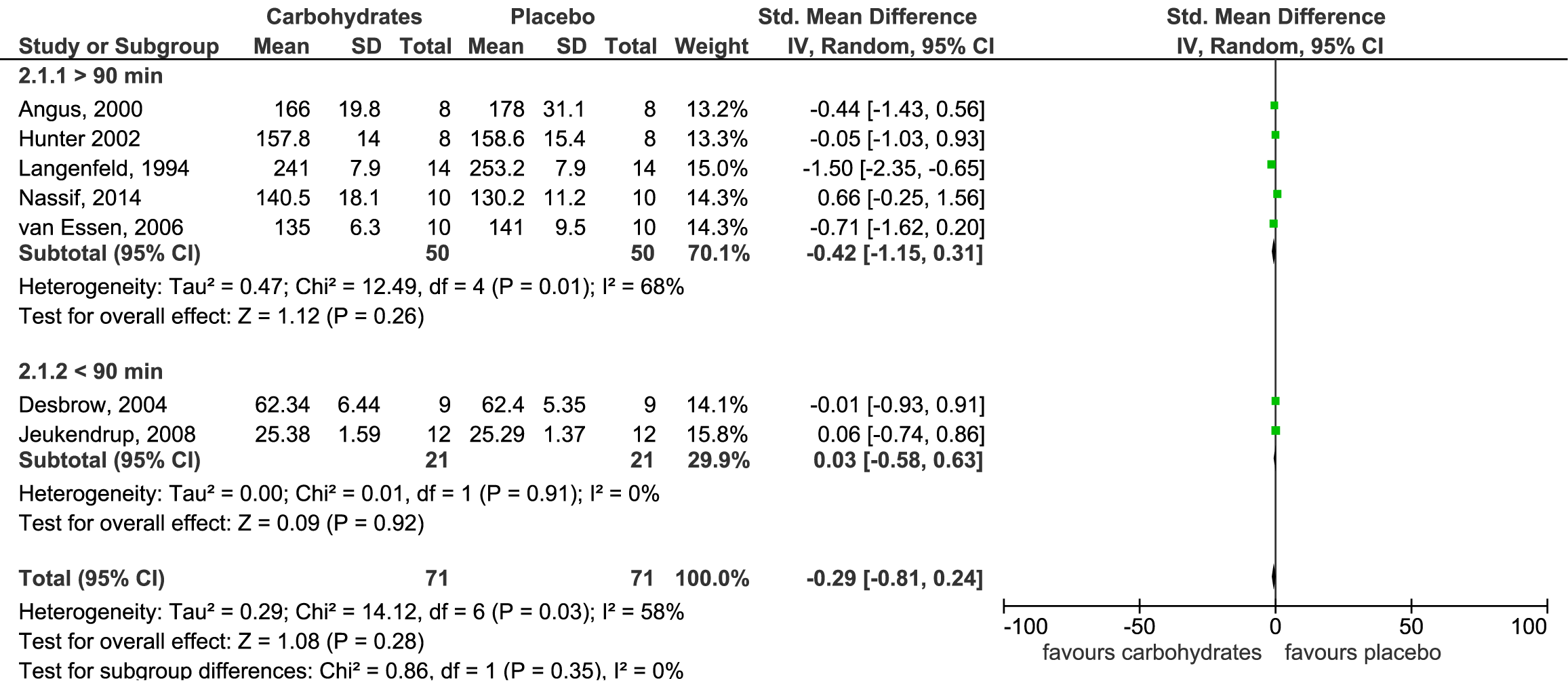

Supplement: Additional file 2: Figure S2. — Effects of carbohydrate interventions as compared to placebo on time required to finish a time trial. Forest plot shows pooled standardized mean differences with 95 % confidence intervals (CI) for 7 randomized controlled trials. Subgroup analyses show the results for exercise duration shorter than 90 min or longer than 90 min, respectively. The diamond at the bottom of the graph and the subgroups represents the pooled mean difference with the 95 % CI for all trials following random effects meta-analyses. (TIF 6850 kb) [file 12970_2016_139_MOESM2_ESM.tif]

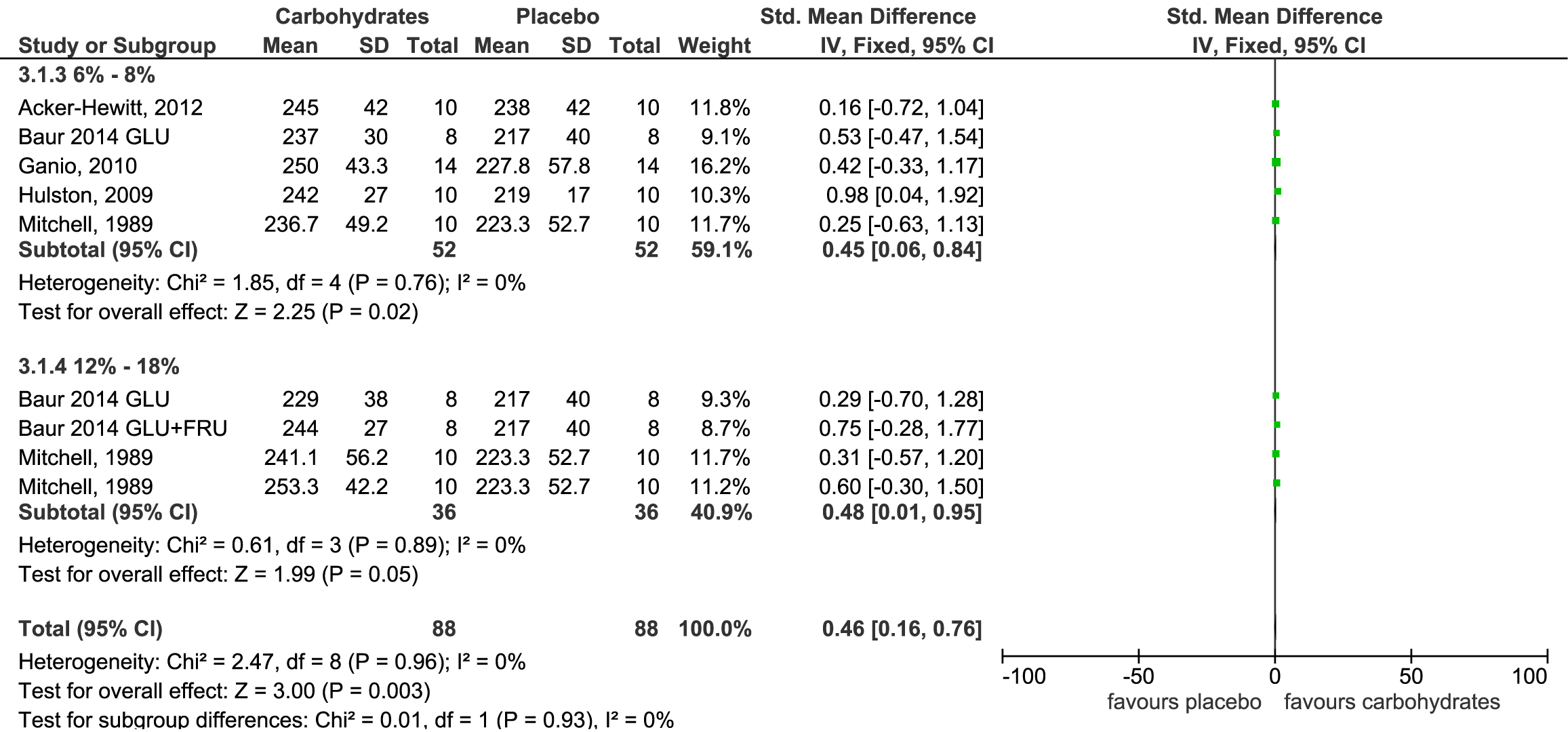

Supplement: Additional file 3: Figure S3. — Effects of carbohydrate interventions as compared to placebo on mean power output. Forest plot shows pooled standardized mean differences with 95 % confidence intervals (CI) for 5 randomized controlled trials. Subgroup analyses show the results for carbohydrate concentrations ranging between 6–8 % and 12–18 %, respectively. The diamond at the bottom of the graph and the subgroups represents the pooled mean difference with the 95 % CI for all trials following fixed effect meta-analyses. GLU = glucose; FRU = fructose. (TIF 7339 kb) [file 12970_2016_139_MOESM3_ESM.tif]

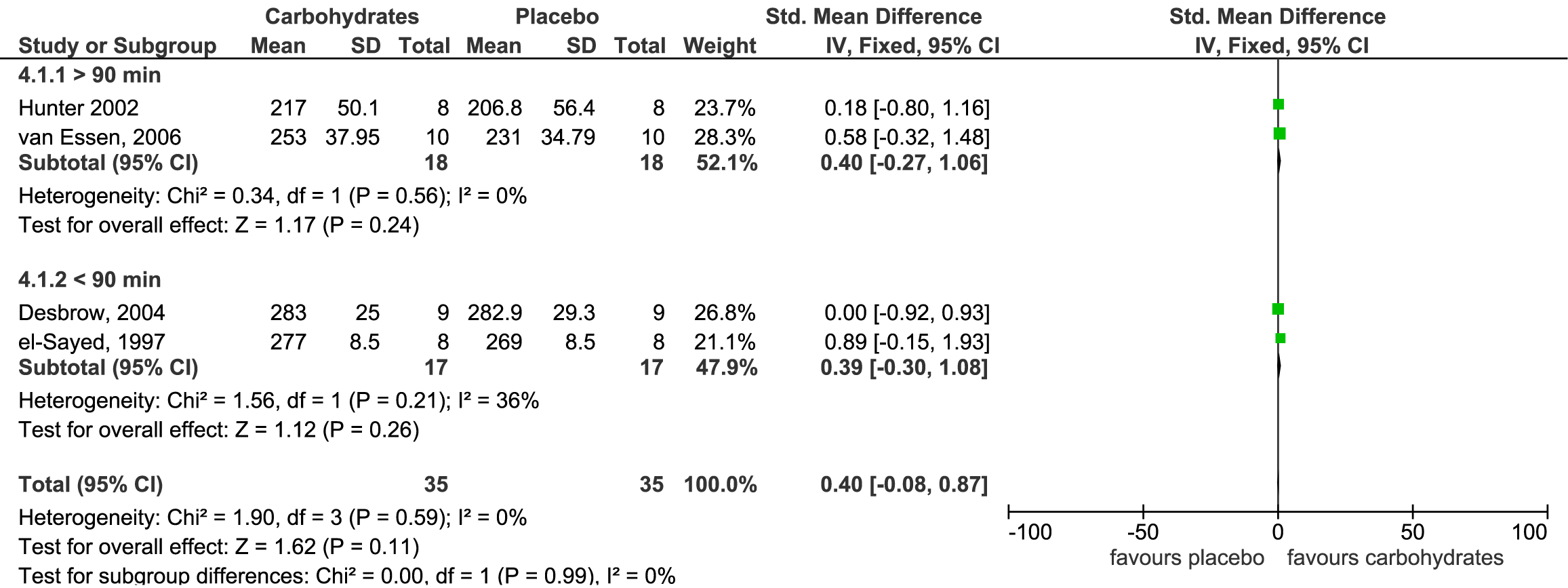

Supplement: Additional file 4: Figure S4. — Effects of carbohydrate interventions as compared to placebo on mean power output. Forest plot shows pooled standardized mean differences with 95 % confidence intervals (CI) for 4 randomized controlled trials. Subgroup analyses show the results for exercise duration shorter than 90 min or longer than 90 min, respectively. The diamond at the bottom of the graph and the subgroups represents the pooled mean difference with the 95 % CI for all trials following fixed effect meta-analyses. (TIF 5891 kb) [file 12970_2016_139_MOESM4_ESM.tif]
